# Profile and needs of primary informal caregivers of older patients in Belgian geriatric day hospitals: a multicentric cross-sectional study

**DOI:** 10.1186/s12877-021-02255-1

**Published:** 2021-05-17

**Authors:** C. Eyaloba, I. De Brauwer, S. Cès, F. Benoit, S. Gillain, L. Pesch, H. Rouvière, S. De Breucker

**Affiliations:** 1 Department of Geriatric Medicine, Erasme Hospital, Université Libre de Bruxelles, 808 Lennikstreet, 1070 Brussels, Belgium; 2 IRSS, Institute of Health and Society, Université Catholique de Louvain, Ottignies-Louvain-la-Neuve, Belgium; 3 Department of Geriatric Medicine, Brugmann Hospital, Université Libre de Bruxelles, Brussels, Belgium; 4 Department of Geriatric Medicine, University Hospital of Liège, Université de Liège, Liège, Belgium

**Keywords:** Primary informal caregiver (PIC), PIC burden, Geriatric day hospital (GDH)

## Abstract

**Background:**

With the improvement of life expectancy, the world faces increasing demands for care of older persons. In this manuscript, we define the characteristics of primary informal caregivers (PIC) of patients aged 75 years and older admitted to geriatric day hospitals (GDH) in Belgium. A PIC is defined as the person who most often provides care and assistance to persons who need to be cared for. We describe PIC socio-demographic characteristics, satisfaction, burden and wishes about caring; the type of assistance provided and received, their self-rated health, socio-demographic and medical characteristics of proxies, in particular the presence of behavioural disorders.

**Methods:**

We conducted a cross-sectional study in 25 GDH.

**Participants:**

Four hundred seventy-five PIC of patients ≥75 years and their proxies. PIC completed a questionnaire at the GDH assessing burden by Zarit Burden Index-12 (ZBI-12), self-rated health, social restriction due to caregiving and financial participation. We compared the characteristics of PIC with high and low burden, and the characteristics of spouses and adult children PIC. We also analyzed factors associated with a high burden in a multivariable logistic regression model.

**Results:**

PIC were mainly women (72%), adult children (53.8%) and spouses (30.6%). The mean age was 64 ± 14 years for PIC and 84 ± 5 years for care recipients. PIC helped for most of Activities in Daily Living (ADL) and Instrumental ADL (iADL). The median ZBI-12 score was 10 [IQR 5–18]. In multivariable regression analysis, a high burden was positively associated in the total group with living with the relative (*p* = 0.045), the difficulty to take leisure time or vacation (*p* <  0.001), behavioral and mood disorders (*p* <  0.001;*p* = 0.005), and was negatively associated with bathing the relative (*p* = 0.017) and a better subjective health status estimation (*p* <  0.001).

**Conclusion:**

Primary informal caregivers, who were predominantly women, were involved in care for ADL and iADL. A high burden was associated with living with the relative, the difficulty to take leisure time or vacation and the relative’s behavioral and mood disorders. Bathing the relative and a subjective health status estimated as good as or better than people the same age, were protective factors against a high burden.

**Supplementary Information:**

The online version contains supplementary material available at 10.1186/s12877-021-02255-1.

## Background

With the improvement of life expectancy, the world faces increasing demands for care of older persons. As other Western countries, Belgium faces increasing demands for the care of older persons due to aging of its population [1]. In January 2019, Belgium had 1.016.804 inhabitants over 75 years old, which accounted for 9% of the population (https://statbel.fgov.be/fr). The main providers of care for older people are frequently primary informal caregivers (PIC) [2–4]. A PIC is defined as the person who most often provides care and assistance without any professional relationship with the person being cared for [5]. We know that PIC are at high risk of exhaustion, stress and adverse outcomes which can result in an undesired care breakdown for the person they care for [6, 7]. A better understanding of their needs is a priority, as defined by the World Health Organization [8]. Recent reviews showed that unmet needs of PIC have to be taken into account in a proactive approach of care planning from health care professionals, and that PIC are often not integrated enough in the health care system [9–11]. In 2011, the Belgian Health Care Knowledge Centre analyzed thirty forms of non-pharmaceutical therapy for people with dementia. Experts applied a level IB of recommendation for multicomponent psychoeducation and psychosocial interventions that have been shown to improve PIC outcomes, like controlling stress, developing strategies for handling their relative’s behavioral problems, reducing their burden and increasing their satisfaction with life. Such interventions also reduce the risk of institutionalization, and improve mood, well-being and quality of life of PIC [12].

The outpatient care is available for geriatric patients in many European countries. In Belgium, as in other countries, older people receive regular care in geriatric day hospital (GDH), an outpatient service funded by the Federal Care Program for geriatric patients. Depending on the hospital and the country, GDH is more oriented to rehabilitation programs or to consultation and comprehensive geriatric assessment. A multidisciplinary team consisting of geriatricians, social workers or community health nurses, nurses, physiotherapists, occupational therapists as well as dieticians, speech therapists and psychologists work in collaboration with general practitioners, and home care services, aiming to provide optimal care for older people and to support them at home for as long as they wish [13, 14]. Based on our experience, despite the well-recognized critical role of social workers, GDH do not currently provide systematically social consultancy to patients, as it is the case for geriatric hospitalized patients, and PIC receive too little attention in outpatient settings [15]. A recent qualitative study in an outpatient geriatric clinic reported that PIC of older people with dementia often receive little or no help, seek information on dementia by internet, and are worried about how their poor health impair their caregiving [16]. We, therefore, aimed to better define the needs of PIC of older patients admitted to GDH, by describing their socio-demographic and psycho-social characteristics, their resources and by the analysis of factors associated with PIC psychological burden for all PIC, and for subgroups of adult children and spouses.

## Methods

The content of the questionnaire was developed with a 2-step approach. First, during a workshop on PIC organized at the 2016 Annual Congress of the Belgian Society of Gerontology and Geriatrics, day-hospital care-workers with a ground expertise in the assessment of frail older people participated in focus groups, aiming to better define the profile and the needs of the PIC of geriatric outpatients and provide them support, in addition to the patient’s specific needs. Four focus groups were organized, namely: who are the PIC and what kind of help is provided by the PIC; how and how often they help, what could be the consequences of helping for PIC, and how GDH teams could support PIC. More details are available on request.

The questionnaire was in a second step completed and finalized by experts in geriatric care working in GDH, with the support of an economist researcher in Public Health specifically involved in primary services evaluation and PIC needs in Belgium, on the basis of the reports of each focus group. Several meetings were organised to choose items to be included in the questionnaire, like validated questionnaires in the literature, and other items, basing on different experts’ advice. The questionnaire should not last more than 30 min and should be comprehensible by as many people as possible. Since the study involved French-speaking GDH, the questionnaire was written in French. The questionnaire was tested by volunteer PIC in the principal investigator’s center (Erasme Hospital) prior to the beginning of the study, in order to ensure the relevance of the questions and the readability of the questionnaire. The questionnaires were distributed to all centers and were explained to at least two members of the participating centers, including the geriatrician in charge of the GDH. If needed, they were allowed to help the PIC to fill out the questionnaire, in order to ensure a maximum of questions answered.

### Participants

The study was carried out in French-speaking Belgium GDH oriented on consultation and diagnostic workup. It aimed PIC of patients aged 75 years and over consulting in participating centers. Of the 34 centers that agreed to participate, 25 provided one or more questionnaires. PIC of patients < 75 years, of hospitalized patients or PIC who were absent during the consultation were excluded. Informal caregivers who were not the primary caregiver were also excluded. All PIC provided a written informed consent.

### Questionnaire

The anonymous questionnaire consisted of 34 questions. Nine questions focused on the PIC’s socio-demographic characteristics: age, gender, nationality, language spoken, level of education, professional status, financial income, and relationship and cohabitation status with the care recipient. Five questions were related to the type of assistance provided, i.e. in basic and/or instrumental activities of daily life (ADLs and/or IADLs,) the frequency and the duration of the assistance, the activities that the caregivers no longer wished to carry out and the assistance provided by other informal or professional caregivers. One question related to the PIC’s self-assessment of his/her health: better, as good as or poorer than that of other people of the same age. One question was related to the satisfaction that one might have to be a PIC. Five questions focused on the individual adverse consequences of caring: the inability to take leisure times, to have time for him/herself for an external social life, how often he/she is interrupted in personal activities during the day, the need to reduce time of work, or the personal financial participation. One question was about the wish for institutionalization of the care recipient. Finally, seven questions were related to the socio-demographic and medical characteristics of the care recipient, including age, gender, the presence of memory and/or mood disorders, behavioral disorders, the acceptance to be assisted in general, and particularly by professional caregivers. Information on care recipients was only delivered by the PIC. Because the questionnaire was anonymous, and the informed consent was addressed to PIC, no additional information from patients’ medical files was possible. At the end of the questionnaire, the PIC could also write free comments.

The PIC’s burden was measured by the Zarit Burden Index-12 Items (ZBI-12) [17], a short form of the Zarit Burden Interview (ZBI) [18]. The higher the score, the higher the caregiver’s burden (total score on 48 points). The score was dichotomized in no or mild burden (score below 13 points) or mild to high burden (score of 13 points or above) according to Gratao et al. [19].

### Data collection

Participants were enrolled between March 1st and December 31, 2018. Five hundred and twenty questionnaires were collected in the 25 GDH, among which 475 were included in the study. Forty-five questionnaires were excluded because the care recipient was under 75 years of age (*n* = 12), or the PIC was not present during the consultation (*n* = 33). The research team interacted regularly with the study centers through regular e-mail newsletters, telephone calls and visited at least once each center to collect the questionnaires. The study was approved by the local ethics committee of each participating center in March 2018 (see declarations above for details on local ethics committees) under the final reference number P2017/574/B406201734547. Erasme Hospital acted as the central ethics committee.

### Statistical analysis

The statistical analyses were carried out using STATA® version 16, Lakeway drive, Texas.

Descriptive statistics were calculated in order to describe characteristics of PIC and care recipients. Results were reported as numbers and percentages for categorical variables, mean ± standard deviation (SD) for normally distributed quantitative variables or median [interquartile range] for quantitative variables with asymmetrical distributions. Normality assessment was based on graphical representations (histogram, box plot).

We performed Student’s t-test, Pearson’s chi-squared or Fischer’s exact test to compare data between groups of PIC with high and low burden. In order to allow an analysis of the most represented groups of PIC, we applied a sub-group analysis to the sample of the study for adult children (≥18 years) and spouses. We compared PIC adult children and spouses for kind of help provided, for help that PIC would no longer wish to do and for perception of PIC regarding the help provided using Pearson’s chi-squared or Fischer’s exact test and Mann-Whitney U test.

Subsequently, we analyzed parameters associated with a high burden (ZBI-12 score ≥ 13) with univariate and multivariable logistic regression analysis for the total group and the two subgroups of adult children and spouses. For the multivariable regression analysis, we used a stepwise regression with a forward selection (*p* = 0.05), a backward selection (*p* = 0.20) and then a mixed approach, considering variables associated with a high burden in univariable regression with a *p*-value inferior to 0.20. We chose in every group the best model based on *p*-values and lowest Akaike information criterion (AIC). We tested goodness of fit with Hosmer-Lemeshow test and verified collinearity between predictor with the variance inflation factor (VIF). Missing data for the variables computed in the multivariable model being considered to be of MAR (Missing at Random) type, we verified that the proportion of missing data was inferior to 10% for each variable and that their distribution was random. We then performed an imputation on missing data using Multiple Correspondence Analysis to verify that the results of our model were similar with the imputed model. The results are expressed in odd ratios and 95% confidence intervals, with the *p*-value from Wald’s test.

## Results

### Characteristics of PIC

Four hundred seventy-five PIC answered the questionnaire. Age ranged from 21 to 90 years, with a mean age of 64 (SD = 14). Most of PIC was women, and the majority was adult children and spouses, following a bimodal distribution of age of respectively 57 ± 8 years and 79 ± 7 years. The majority of the PIC had at least a middle school degree, and only a marginal fraction of PICs was illiterate. Fifty-four percent of PIC had a monthly income equal or greater than 2000 Euros, and a small percentage (3%) were in a financially precarious situation, with an income of less than 1000 Euros per month. Twelve percent of PIC helped their relative financially, and one in five participated in the cost of home care support, with a monthly average of 304 Euros. A third of PIC was professionally active. Of these, 79% were adult children; of whom 13% had to adapt their working hours to help their parent.

### Care recipients’ characteristics

The majority of care recipients (98%, *n* = 465) lived at home, 2% lived in a nursing home. Their mean age was 84 (SD = 5, min 21-max 94) years old, 66% were women. Overall, 74% had memory impairment, 39% mood disorders, and 21% behavioral disorders. Most of them accepted help from relatives (80%) and professionals (70%). Memory impairment, mood disorder and/or behavioral disorder and reluctance to help of the care recipients were significantly more frequent in the group of PIC with a high burden (all *p* <  0.001).

The characteristics of the PIC and the care recipients are summarized in Table 1.

**Table 1:** Characteristics of PIC and care recipients in total, high and low burden groups

Caregivers characteristics	n	Total (*n* = 475)	ZBI-12 ≥ 13 (*n* = 181)	ZBI-12 < 13 (*n* = 270)	*p*-value
***Age*** (mean ± SD)					
Total	474	64 ± 14	63 ± 12	63 ± 14	0.741
Adult children	255	57 ± 8	57 ± 7	57 ± 8	0.651
Spouses	145	79 ± 7	77 ± 7	80 ± 6	**0.037**
***Gender (females)*** n (%)	475	342 (72)	131 (77)	185 (69)	0.072
***CR relationship*** n (%)	474				0.421
Child		255 (54)	102 (57)	145 (54)	
Spouse		145 (31)	57 (32)	74 (28)	
Friend		17 (4)	3 (2)	12 (4)	
Nephew/Niece		12 (3)	5 (3)	6 (2)	
Sibling		3 (1)	1 (1)	2 (1)	
Neighbour		2 (0.5)	0	2 (1)	
Other		40 (8)	12 (7)	27 (10)	
***Educational level*** n (%)	459				0.102
Illiterate		3 (1)	0 (0)	3 (1)	
Primary school		44 (9.5)	10 (6)	24 (10)	
Secondary school		108 (23.5)	40 (23)	62 (24)	
High school degree		143 (31)	55 (31)	85 (33)	
Higher education		115 (25)	54 (31)	54 (21)	
University degree		46 (10)	17 (10)	28 (11)	
***Professional Status*** n (%)	470				0.238
Retired		210 (45)	69 (39)	123 (46)	
Active		159 (34)	72 (40)	84 (32)	
Not active (at home, invalid, or unemployed)		93 (20)	34 (19)	54 (20)	
Other		8 (2)	4 (2)	4 (1.5)	
***Monthly financial income level in Euros*** n (%)	386				0.247
< 500		2 (0.5)	0 (0)	2 (1)	
500–1000		8 (2)	3 (2)	5 (2)	
1000–1500		87 (23)	36 (23)	49 (23)	
1500–2000		82 (21)	29 (18)	49 (23)	
2000–2500*		73 (19)	35 (22)	34 (16)	
2500–3000*		56 (15)	28 (18)	26 (12)	
> 3000*		78 (20)	27 (17)	50 (23)	
***Living with the care recipient*** n (%)	464				
Total group		201 (43)	86 (48)	104 (39)	0.063
Children		52 (21)	27 (27)	24 (17)	0.068
Spouses		128 (91)	51 (93)	67 (91)	0.758
***Number of persons assisted*** median [IQR]	455	1 [1–10]*	1 [1–10]	1 [1–10]	0.682
**Care recipients’ characteristics**					
***Age in years*** (mean ± SD)	474	84 ± 5	84 ± 5	84 ± 5	0.144
***Gender (female)*** n (%)	473	314 (66)	119 (66)	184 (68)	0.652
***Memory impairment*** n (%)	452	333 (74)	146 (84)	171 (66)	< 0.001
***Mood disorder*** n (%)	452	178 (39)	106 (62)	63 (24)	< 0.001
***Behavioral disorder*** n (%)	452	97 (21)	66 (38)	26 (10)	< 0.001
***Acceptance of aid in general*** n (%)	408	325 (80)	112 (68)	204 (88)	< 0.001
***Acceptance of professional help*** n (%)	416	293 (70)	90 (56)	195 (81)	< 0.001

*PIC* Primary Informal Caregivers - ZBI-12: Zarit Burden Index – *[min 1-max 13]

### PICs’ activities

The median duration of assistance was 5 [2 – 10] years. Almost half (43%) of the PIC lived with the care recipient; among them, 64% were spouses and 26% were adult children. The PICs were involved in care for basic activities of daily living (ADL), but more for instrumental ADLs (IADL). Adult children were more involved in transportation for obligatory tasks and help organization, and spouses were more in charge of meal preparation, managing medication and doing household chores (Table 2). Incontinence management, bathing, medication management and household chores were the main activities that the PIC no longer wished to do. Adult children wished more often to stop feeding their relative and manage medication than spouses. Only 15% of the PICs participated in care education programs.

**Table 2:** Help provided by PIC (total group, adult children and spouses subgroups) and professional caregivers, and help that PIC would no longer wish to do

	Kind of help provided					Professional help	Help that PIC would no longer wish to do			
	n	Total group (***n*** = 475)	Adult children (***n*** = 255)	Spouses (***n*** = 145)	***p***-value		Total group (*n* = 475)	Adult children (***n*** = 255)	Spouses (***n*** = 145)	***p***-value
**Basic ADLs**										
Toileting	475	189 (39)	94 (37)	64 (44)	0.152	226 (47)	25 (13)	13 (14)	8 (13)	0.789
Bathing	475	182 (38)	95 (37)	64 (44)	0.176	215 (45)	29 (16)	15 (16)	11 (17)	0.861
Dressing	475	192 (41)	104 (41)	64 (44)	0.514	125 (26)	22 (11)	13 (13)	7 (11)	0.789
Eating	475	201 (42)	103 (40)	68 (47)	0.206	85 (18)	26 (13)	17 (16)	4 (6)	**0.036**
Incontinence management	475	197 (41)	104 (41)	63 (43)	0.604	143 (30)	50 (26)	31 (31)	14 (23)	0.279
**Instrumental ADLs**										
Indoor Mobility	475	194 (41)	106 (42)	58 (40)	0.759	87 (18)	24 (13)	13 (12)	7 (13)	0.983
Meal preparation	473	266 (56)	133 (53)	100 (69)	**0.001**	120 (25)	33 (12)	19 (15)	12 (12)	0.585
Laundry	475	307 (64)	159 (62)	101 (70)	0.141	120 (25)	47 (16)	32 (21)	12 (12)	0.074
Household chores	475	263 (55)	137 (54)	93 (64)	**0.043**	246 (52)	65 (25)	41 (31)	23 (25)	0.317
Medication management	475	266 (56)	132 (52)	97 (67)	**0.003**	198 (42)	50 (19)	36 (27)	10 (10)	**0.002**
Finance management	475	366 (76)	206 (81)	108 (74)	0.140	46 (10)	18 (5)	10 (5)	5 (5)	0.950
Transport for leisure	475	322 (68)	176 (69)	89 (62)	0.143	67 (14)	25 (8)	17 (10)	5 (6)	0.260
Transport for obligatory tasks	475	389 (82)	225 (89)	102 (70)	**< 0.001**	81 (17)	38 (10)	26 (12)	7 (7)	0.192
Shopping	475	352 (74)	187 (74)	105 (72)	0.793	78 (16)	37 (11)	25 (14)	8 (8)	0.136
Help organisation	475	343 (72)	194 (76)	97 (67)	**0.047**	71 (15)	35 (10)	21 (11)	10 (10)	0.882

*ADL* Activities of Daily Living, *PIC* Primary Informal Caregivers. Results are expressed in n (%)

### PIC’s burden and experiences

Most PIC (83%) felt satisfied in caring for their relative and didn’t want him/her to go in a nursing home. Two thirds estimated that their health status was similar to that of other people of their age. Almost half of the PICs found that they have enough time for themselves, while 12% stated that they never have time for themselves, and particularly spouses. Spouses were also more interrupted in activities every day than adult children (19% versus 7%), and were also more interrupted at night, from several times a week to several times at night. Adult children were more prone to wish to place their relative in a nursing home than spouses 22 versus 10%). (Table 3).

**Table 3:** Perception of PIC regarding the help provided

Characteristics	n	Total group (***n*** = 475)	Adult children (***n*** = 255)	Spouses (***n*** = 145)	***p***-value
***Satisfaction in caring for care recipient***	442	367 (83)	188 (81)	117 (86)	0.191
***Self-rated Health***	456				0.385
Poorer		93 (20)	52 (21)	26 (19)	
As good		310 (68)	166 (68)	89 (65)	
Better		53 (12)	26 (11)	21 (15)	
***Having time for him/herself***	439				**0.001**
Never		52 (12)	21 (9)	24 (19)	
Insufficient		176 (40)	96 (40)	58 (46)	
Enough		211 (48)	126 (52)	44 (35)	
***Difficulty to take leisure time***	441	158 (36)	91 (38)	43 (33)	0.367
***Interruption of activity during the day***	427				**< 0.001**
Never		233 (55)	139 (59)	53 (42)	
Several times a week		120 (28)	71 (30)	29 (23)	
Every day		44 (10)	17 (7)	24 (19)	
Several times a day		30 (7)	10 (4)	19 (15)	
***Disturbance at night***	413				**< 0.001**
Never		332 (81)	193 (85)	80 (69)	
Several times a week		63 (15)	30 (13)	25 (22)	
Every night		11 (3)	1 (0.5)	7 (6)	
Several times at night		6 (2)	2 (1)	4 (3)	
***Wish to place the proxy in a nursing home***	431	73 (17)	49 (22)	14 (10)	**0.004**
***Zarit Burden Index – 12 items***	451	10 [5–18]	11 [6–19]	11 [4–19]	0.677

*PIC* Primary Informal Caregivers. Results are expressed in median [IQR] and n (%)

The median ZBI-12 was 10 [5 - 18], and 40% had a ZBI-12 score above or equal to 13 points, indicating a high burden. The burden score didn’t differ between adult children and spouses (Table 1).

Factors significantly associated with a high burden in univariate analysis are summarized in Table 4.

**Table 4:** Analysis of factors associated with Zarit Burden Index (12 items) in univariate logistic regression

Characteristics	Total group (***n*** = 451; ZBI≥13 = 181)			Adult children (***n*** = 247; ZBI≥13 =102)			Spouses (***n*** = 131; ZBI≥13 =57)		
	n	OR (95% CI)	***p***-value	n	OR (95% CI)	***p***-value	n	OR (95% CI)	***p***-value
**PIC characteristics**									
Age	448	0.99 (0.98 - 1.01)	0.741	246	0.99 (0.96 - 1.02)	0.649	131	0.94 (0.89 - 0.99)	**0.041**
Gender (F)	449	0.67 (0.44 - 1.04)	0.073	247	0.62 (0.33 - 1.16)	0.133	131	0.53 (0.26 - 1.08)	0.080
Level of education^a^	432		0.126	238		0.247	123		0.363
Primary school		1.00			1.00			1.00	
Secondary school		1.74 (0.76 – 3.98)			1.38 (0.32 – 5.98)			2.23 (0.73 – 6.85)	
High school degree		1.75 (0.78 – 3.89)			1.96 (0.49 – 7.90)			1.30 (0.38 – 4.43)	
Higher education		2.70 (1.19 – 6.12)			2.92 (0.71 – 12.02)			3.03 (0.86 – 10.72)	
University degree		1.64 (0.64 – 4.21)			1.52 (0.34 – 6.86)			1.75 (0.30 – 10.34)	
Professional status	444		0.247	244		0.068	130		0.823
Retired		1.00			1.00			1.00	
Active		1.53 (0.99 – 2.35)			2.25 (1.18 – 4.29)			0.67 (0.06 – 7.58)	
Not active		1.12 (0.67 – 1.89)			1.35 (0.63 – 2.92)			1.20 (0.45 – 3.20)	
Other		1.78 (0.43 – 7.35)			2.37 (0.31 – 18.07)			2.67 (0.23 – 30.33)	
Living with the relative	442	1.44 (0.98 - 2.11)	0.064	242	1.78 (0.95 - 3.31)	0.070	129	1.33 (0.37 - 4.80)	0.661
**PIC perception of care**									
Tasks performed by PIC:									
Toileting	451	0.67 (0.45 - 0.98)	**0.041**	247	0.57 (0.33 - 0.98)	**0.043**	131	0.56 (0.28 - 1.14)	0.110
Bathing	451	0.66 (0.44 - 0.97)	**0.037**	247	0.63 (0.37 - 1.07)	0.086	131	0.60 (0.30 - 1.20)	0.148
Dressing	451	1.13 (0.78 - 1.66)	0.516	247	0.90 (0.54 - 1.51)	0.688	131	1.65 (0.82 - 3.32)	0.157
Preparing meals	447	1.40 (0.95 - 2.06)	0.090	244	1.12 (0.68 - 1.88)	0.644	130	2.09 (0.92 - 4.72)	0.077
Laundry	451	1.17 (0.78 - 1.74)	0.441	247	1.03 (0.61 - 1.74)	0.914	131	1.89 (0.83 - 4.29)	0.130
Mandatory transports	450	1.77 (1.04 - 3.04)	**0.037**	246	1.48 (0.63 - 3.43)	0.366	131	1.89 (0.83 - 4.29)	0.130
Finance management	451	1.31 (0.82 - 2.10)	0.250	247	0.69 (0.36 - 1.31)	0.254	131	3.83 (1.44 - 10.20)	**0.007**
Organization of home help	451	2.06 (1.31 - 3.25)	**0.002**	247	2.65 (1.36 - 5.16)	**0.004**	131	1.26 (0.59 - 2.72)	0.552
Supervision of health care	268	1.92 (1.15 - 3.20)	**0.013**	170	2.26 (1.16 - 4.43)	**0.017**	131	0.99 (0.34 - 2.85)	0.976
PIC education	274	1.71 (0.88 - 3.34)	0.114	173	1.47 (0.59 - 3.67)	0.407	62	1.01 (0.27 - 3.76)	0.983
The satisfaction to be a caregiver	423	0.61 (0.37 - 1.02)	0.061	228	0.53 (0.27 - 1.03)	0.063	126	0.57 (0.21 - 1.56)	0.275
The wish to institutionalize the relative	415	3.49 (2.02 - 5.99)	**< 0.001**	221	3.78 (1.91 - 7.45)	**< 0.001**	128	2.83 (0.81 - 9.95)	0.104
Difficulty to take leisure time or vacation	423	8.10 (5.17 – 12.71)	**< 0.001**	235	5.79 (3.26 - 10.31)	**< 0.001**	120	11.39 (4.54 - 28.57)	**< 0.001**
Need to lower professional time schedule	425	4.42 (2.53 - 7.73)	**< 0.001**	238	4.56 (2.13 - 9.73)	**< 0.001**	116	2.79 (1.05 - 7.38)	**0.039**
Need to help financially the proxy	439	2.59 (1.44 - 4.66)	**0.002**	242	2.67 (1.20 - 5.93)	**0.016**	125	1.06 (0.39 - 2.90)	0.908
Subjective health status estimation	439		**< 0.001**	239		**< 0.001**	128		**0.005**
Poorer		1.00			1.00			1.00	
As good		0.19 (0.11 – 0.32)			0.19 (0.10 – 0.39)			0.17 (0.05 – 0.61)	
Better		0.14 (0.06 – 0.30)			0.15 (0.05 – 0.43)			0.24 (0.09 – 0.65)	
**Care recipients’ characteristics**									
Cognitive disorders	434	2.71 (1.08 – 3.86)	**< 0.001**	240	2.05 (1.08 - 3.86)	**0.027**	125	1.93 (0.82 – 4.53)	0.132
Behavioral disorders	433	5.55 (3.34 - 9.22)	**< 0.001**	243	4.18 (2.20 - 7.92)	**< 0.001**	121	4.87 (1.86 - 12.75)	**0.001**
Mood disorders	433	5.04 (3.32 - 7.67)	**< 0.001**	241	4.33 (2.50 - 7.51)	**< 0.001**	121	5.45 (2.46 - 12.04)	**< 0.001**
Refusal of help	396	3.38 (2.02 - 5.65)	**< 0.001**	224	3.25 (1.65 – 6.43)	**0.001**	110	4.04 (1.44 - 11.32)	**0.008**

^a^illiterate omitted (n too small)

Using a multivariable logistic regression analysis, we observed that burden in the total group depended on living with the relative (Adj OR 1.75 [1.01–3.04]), bathing the relative (Adj OR 0.50 [0.28–0.88]), the difficulty to take leisure time or vacation (Adj OR 8.28 [4.77–14.37]), a subjective health status estimated as good (Adj OR 0.16 [0.06–0.44]) or better (Adj OR 0.18 [0.09–0.35]) than people of the same age, and the relative’s behavioral and mood disorders (Adj OR 3.63 [1.75–7.53] and 2.39 [1.31–4.38]). In the adult children group specifically, a high burden did not significantly depend on living with or bathing the relative. In the spouse group too, living with or bathing the relative and behavioral disorders were not significantly associated with the burden (Table 5).

**Table 5:** Analysis of factors independently associated with Zarit Burden Index (12 items) in multivariable logistic regression

Characteristics	Total group (***n*** = 388; ZBI ≥ 13 = 156)		Adult children (***n*** = 225; ZBI ≥ 13 = 91)		Spouses (***n*** = 111; ZBI ≥ 13 = 50)	
	OR_**adjusted**_ (95% CI)	***p***-value	OR_**adjusted**_ (95% CI)	***p***-value	OR_**adjusted**_ (95% CI)	***p***-value
**PIC characteristics**						
Living with the relative	1.75 (1.01–3.04)	**0.045**	–		–	
Toileting	-		0.51 (0.25–1.07)	**0.077**	-	
Bathing	0.50 (0.28–0.88)	**0.017**	-		-	
Difficulty to take leisure time or vacation	8.28 (4.77–14.37)	**< 0.001**	7.44 (3.68–15.05)	**< 0.001**	8.04 (2.93–22.02)	**< 0.001**
Subjective health status estimation		**< 0.001**		**< 0.001**		
Poorer	1.00		1.00		1.00	0.173
As good	0.16 (0.06–0.44)		0.20 (0.06–0.69)		0.31 (0.07–1.48)	
Better	0.18 (0.09–0.35)		0.17 (0.08–0.40)		0.34 (0.07–1.10)	
**Care recipients’ characteristics**						
Behavioral disorders	3.63 (1.75–7.53)	**< 0.001**	3.45 (1.40–8.53)	**0.007**	-	
Mood disorders	2.39 (1.31–4.38)	**0.005**	2.43 (1.13–5.23)	**0.023**	4.70 (1.81–12.19)	**0.001**

## Discussion

Although our study focused only on PIC of outpatient in GDH, our findings are consistent with those of other international and Belgian studies: most PIC are women and often providing care without outside help for ADLs as well as for IADLs [1, 2, 4, 14, 20–22], and this care has a financial cost for PIC [4, 23–25]. We can identify from this survey the profile of the PIC in Belgium and the factors associated with caregiver’s psychological burden. One in four PIC has an income between 1000 and 1500 Euros per month, which is low compared to the standard of living in Belgium (the minimal social integration income for a single individual is 958, 91 Euros per month (https://www.mi-is.be/fr/outils-cpas/montantsrevenusminimum).

Fifteen percent of active PIC, mainly adult children, has to adjust their working hours. PIC are more involved in care for IADLs and much more often than professionals. A high Zarit-12 score is associated with several ADLs and IADLs tasks in univariate logistic regression analysis, indicating that professional help would be beneficial to caregivers. This observation reinforces the findings from a previous study that reported the need to strengthen professional home help for IADLs and ADLs [14] and from other studies. A stronger association was observed between PICs’ burden and the caregiving tasks than with the characteristics of the care recipient, except when the care recipients had dementia and behavioral problems [21, 26–28]. The subjective health assessment is a powerful and useful criterion to determine health status, and its association with a higher risk of burden was described previously [29–31]. More specifically, PIC of patients with behavioral or mood disorders should systematically get adequate support and appropriate knowledge on the pathology causing the disorder and on its development in terms of prognosis [28, 32, 33]. An attention should be paid to PIC living with the care recipient with behavioral or mood disorders while also considering the particular needs of support of children and spouses PIC [34]. Tailored-activities programs combining psycho-educational support for PIC and assessment of abilities and interests of patients have shown mild to moderate effect on caregiver burden in meta-analyses, with promising non-pharmacological solutions to prevent PICs’ burden [9, 35]. In addition, developing respite services to support leisure activities for PICs could prevent burden. Regarding the difficulties of accepting help, psychological support should also be provided to the patient to promote acceptance of familial and professional help.

A recent review on the health effects of caregiving summarized negative and positive effects of caregiving. Caring was described as a mixture of satisfaction and represented a challenge for the PIC, making it crucial to pay attention to the PICs’ health status [36–38].

We observed that while caregiving can be stressful, it can also be rewarding: 83% of PIC were satisfied to take care of their relatives and did not wish to place them in a nursing home, suggesting that the assistance provided strengthens family ties and the feeling of satisfaction of taking care [39]. For PIC who wish to place their relative in nursing home, they should benefit in priority from an individual follow-up since it reveals a situation at risk for both PIC, for mental and health status and care recipient, for elder abuse and unmet needs. Moreover, in Belgium, it may take several months to find a place in nursing home. On the contrary, PIC could feel guilty for having to put their proxy in a nursing home [31, 40]. It is worth keeping in mind, however, that our survey does not cover all the factors that could influence PICs’ burden, and that the reality is much more complex. The refusal of professional help or a placement in a nursing home are sometimes underpinned by a feeling of guilt from the PIC, or by a feeling of failure, which could have influenced the answers. GDH could play a supporting and accompanying role in preventing the deterioration of the caregiver’s health, particularly of spouses, who are often as old as the care recipients.

Many PIC do not know to whom or where to turn when they need professional help. This study suggests that geriatric outpatient teams can play a major role in identifying the needs of PIC in GDH, focusing first on PIC at risk of high burden. It could allow providing early social and psychological assistance, continuing education on chronic diseases and dementia, in collaboration with general practitioners and dedicated non-profit organizations, while taking into account the individual risk factors of both the PIC and the care recipients.

### Strengths and limitations of the study

We have carried out a cross-sectional study on 25 GDH. To our knowledge, this is the largest study carried out on PIC in GDH. As the questionnaire was based on validated tools together with questions from experts in the field of geriatric care and public health, it has not received validation before, and cannot be used for further studies unless validated. As this survey was limited to patients attending the GDH, our results cannot be extrapolated to the general population. Also, we do not have evidence that our sample is fully representative of the ICPs in the centers studied.

In addition, unlike many other studies, our study was not focused only on demented patients, and information on care recipients was only provided by PIC and might have been distorted.

Furthermore, patients with severely reduced mobility who no longer attend GDH are probably underrepresented. Despite the good cooperation of the centers, we had to exclude PIC of patients who were not accompanied by them. It was not always easy to identify the PIC. This led to potential selection bias. Adult children represented the largest number of PIC interviewed. Because they are more involved in mandatory mobility assistance, it is likely that elderly spouses who are PIC did not attend the GDH consultation with their partner. In addition, our study was only dedicated to PIC, which limited the number of participants.

## Conclusion

This study shows that primary informal caregivers of older patients attending assessment in geriatric day hospitals are mainly women, and are mainly adult children or spouses. They are frequently involved in ADL and IADL and they feel burdened. A high burden was associated with living with the relative, the difficulty to take leisure time or vacation and the relative’s behavioral and mood disorders. Bathing the relative and a subjective health status estimated as good as or better than people the same age were protective factors against a high burden. Although our study focused only on caregivers of outpatients in GDHs, our findings are consistent with those reported in the literature and highlight the importance of financial and psychosocial impacts of helping his/her relative.

## Supplementary Information

**Additional file 1.** Questionnaire for Primary informal Caregivers at the geriatric day hospital: a survey in French-speaking Belgium centers

## Data Availability

The data sets generated and analyzed during the current study are not publicly available due to the need of approval from each local investigator before sharing, but they are available from the corresponding author on reasonable request.

## Abbreviations

PIC: Primary informal caregiver
GDH: Geriatric day hospital
ZBI-12: Zarit Burden Index-12 Items
ADLs: Basic Activities of Daily Living
IADLs: Instrumental Activities of the Daily Living
