# Supplementary material for: Profile and needs of primary informal caregivers of older patients in Belgian geriatric day hospitals: a multicentric cross-sectional study
Source: BMC Geriatr. 2021 May 17;21:315. doi: 10.1186/s12877-021-02255-1 (PMC8130294; doi:10.1186/s12877-021-02255-1)
Supplement: Supplementary file 1 — Additional file 1. Questionnaire for Primary informal Caregivers at the geriatric day hospital: a survey in French-speaking Belgium centers [file 12877_2021_2255_MOESM1_ESM.pdf]

Additional file 1: questionnaire

Profile and needs of primary informal caregivers of older patients in Belgian geriatric day hospitals: a multicentric cross-sectional study.

Authors and affiliations:

Eyaloba Cécile, Department of Geriatric Medicine, Erasme Hospital, Université Libre de Bruxelles
De Brauwer Isabelle, [†]IRSS, Institute of Health and Society, Université Catholique de Louvain
Cès Sophie, [†]IRSS, Institute of Health and Society, Université Catholique de Louvain
Benoit Florence, Department of Geriatric Medicine, Brugmann Hospital, Université Libre de Bruxelles

Gillain Sophie, Department of Geriatric Medicine, University hospital of Liège, Université de Liège

Pesch Laurie, Department of Geriatric Medicine, Erasme Hospital, Université Libre de Bruxelles
Rouvière Héloise, Department of Geriatric Medicine, Erasme Hospital, Université Libre de Bruxelles
De Breucker Sandra, Department of Geriatric Medicine, Erasme Hospital, Université Libre de Bruxelles

From the *Department of Geriatric Medicine, Erasme Hospital, Université Libre de Bruxelles; [†]IRSS, Institute of Health and Society, Université Catholique de Louvain; [‡]Department of Geriatric Medicine, Brugmann Hospital, Université Libre de Bruxelles; and [§]Department of Geriatric Medicine, University hospital of Liège, Université de Liège

Madam, Sir,

This questionnaire is intended for you as a caregiver of a person being cared for in a geriatric day hospital center. By answering our questions, you will help us to better identify the needs of family caregivers and we thank you for this.

1. **Are you the person who spends the most time to helping the loved one who is now consulting in the geriatric day hospital?**

    - o Yes
    - o No

If so, could you answer the questions below? This will take you about 10 to 15 minutes.

2. **How old are you (in years)?**

3. **Are you a woman or a man?**

    - o Woman
    - o Man

4. **What is the relationship or proximity to the person being helped?**

    ☐ Spouse
    ☐ Child
    ☐ Friend
    ☐ Cousin
    ☐ Neighbor
    ☐ Sister or brother
    ☐ Nephew
    ☐ Other: please, specify

5. **What is your professional status?**

    ☐ Employed
    ☐ Self-employed
    ☐ Student
    ☐ Retired
    ☐ Rentier
    ☐ Time credited
    ☐ Unemployed
    ☐ Housewife
    ☐ Invalid: sick leave / career break / parental leave / palliative leave (please circle)

6. **What is your level of education?**

☐ Not attended school
☐ Primary school
☐ Lower secondary education
☐ Higher secondary education
☐ Non-university higher education
☐University higher education

7. **What is your country of origin?**

8. **What is your nationality?**

9. **What is the language spoken with your relative?**

10. **How long have you been caring for your relative?**

11. **Is this the only person you regularly help (considering children, grandchildren, ...)?**

12. **Is there another non-professional caregiver taking care of your family member?**

o Yes
o No

13. **Do you live with the person being helped?**

o Yes
o No

14. **If you do not live with the person you are caring for, how often do you have to visit your relative's home?**

☐ Every day
☐ 1 - 2 times per week
☐ 3 - 4 times per week
☐ 5 - 6 times per week
☐ Other frequency

15. **Do you have a vehicle?**
o Yes
o No

16. **What is the level of your monthly net household income?**
☐ <500 Euros
☐ 500-1000 Euros
☐ 1000-1500 Euros
☐ 1500-2000 Euros

☐ 2000-2500 Euros
☐ 2500-3000 Euros
☐ >3000 Euros
☐ I don't know

17. Here is a table showing the possible help you can give to your loved one, please complete it.

SMALL DAILY TOILET: SHAVING / HAIR STYLING / BRUSHING TEETH, ...	
If you do this activity, is it an activity you wish no longer to do?	○ Yes, I would like to stop doing this activity.<br>○ No
If external assistance is provided, what type of professional provides the assistance?	○ House keeper<br>○ Nurse<br>○ Other: ………………………………………..
BATH/SHOWER	
If you do this activity, is it an activity you wish no longer to do?	○ Yes, I would like to stop doing this activity.<br>○ No
If external assistance is provided, what type of professional provides the assistance?	○ House keeper<br>○ Nurse<br>○ Other: ………………………………………..
ELIMINATION OF URINE AND FAECES<br>TOILET USE, INCONTINENCE MANAGEMENT, MOBILITY AIDS, WIPING AFTER DISPOSAL, PUTTING ON/REMOVING PADS, CLEANING EQUIPMENT	
If you do this activity, is it an activity you wish no longer to do?	○ Yes, I would like to stop doing this activity.<br>○ No
If external assistance is provided, what type of professional provides the assistance?	○ House keeper<br>○ Nurse<br>○ Other: ………………………………………..
FOOD: SERVING DISHES / PUTTING FOOD IN YOUR MOUTH	
If you do this activity, is it an activity you wish no longer to do?	○ Yes, I would like to stop doing this activity.<br>○ No

If external assistance is provided, what type of professional provides the assistance?	o House keeper<br>o Nurse<br>o Other: ………………………………………..
MOBILITY AID INSIDE THE HOME: TRANSFERS TO BED AND/OR CHAIR AND/OR WALKING INSIDE THE HOME	
If you do this activity, is it an activity you wish no longer to do?	o Yes, I would like to stop doing this activity.<br>o No
If external assistance is provided, what type of professional provides the assistance?	o House keeper<br>o Nurse<br>o Other: ………………………………………..
UNDRESSING/DRESSING	
If you do this activity, is it an activity you wish no longer to do?	o Yes, I would like to stop doing this activity.<br>o No
If external assistance is provided, what type of professional provides the assistance?	o House keeper<br>o Nurse<br>o Other: ………………………………………..
MEAL PREPARATION: PREPARING FOOD (WASHING, PEELING, CUTTING, ETC.), HEATING FOOD OR LIQUIDS, SETTING THE TABLE, ETC.	
If you do this activity, is it an activity you wish no longer to do?	o Yes, I would like to stop doing this activity.<br>o No
If external assistance is provided, what type of professional provides the assistance?	o House helper<br>o House keeper<br>o Other: ………………………………………..
SHOPPING: GOING TO THE SHOPS, PAYING AND RETURNING, PUTTING AWAY THE GROCERIES	
If you do this activity, is it an activity you wish no longer to do?	o Yes, I would like to stop doing this activity.<br>o No
If external assistance is provided, what type of professional provides the assistance?	o House keeper<br>o Nurse<br>o Other: ……………………………….

FINANCIAL MANAGEMENT: Paying bills, managing bank account/legal affairs/insurance, ...	
If you do this activity, is it an activity you wish no longer to do?	o Yes, I would like to stop doing this activity.<br>o No
If external assistance is provided, what type of professional provides the assistance?	o House keeper<br>o Administrative tutor<br>o Other: …………………………………………
**HOUSEHOLD: CLEANING THE HOME, WASHING DISHES, DUSTING, TIDYING UP THINGS, WASHING FLOORS, WINDOWS…**	
If you do this activity, is it an activity you wish no longer to do?	o Yes, I would like to stop doing this activity.<br>o No
If external assistance is provided, what type of professional provides the assistance?	o House helper<br>o House keeper<br>o Other: …………………………………………
**LAUNDRY AND IRONING**	
If you do this activity, is it an activity you wish no longer to do?	o Yes, I would like to stop doing this activity.<br>o No
If external assistance is provided, what type of professional provides the assistance?	o House helper<br>o House keeper<br>o Other: …………………………………………
**TRANSPORT FOR MEDICAL FOLLOW-UP OR FOR ADMINISTRATIVE MATTERS**<br>USE OF ANY TYPE OF TRANSPORT (PUBLIC OR PERSONAL VEHICLE) OR MOBILITY AID (WITH OR WITHOUT EQUIPMENT), INCLUDING THE TIME OF THE APPOINTMENT IF YOU ASSIST THE PERSON DURING THE APPOINTMENT OR IF YOU WAIT FOR HIM/HER.	
If you do this activity, is it an activity you wish no longer to do?	o Yes, I would like to stop doing this activity.<br>o No
If external assistance is provided, what type of professional provides the assistance?	o House keeper<br>o Other: …………………………………………

TRANSPORT FOR LEISURE ACTIVITY (FRIENDS, FAMILY, ...)<br>USE OF ANY TYPE OF TRANSPORT OR MOBILITY AID (WITH OR WITHOUT EQUIPMENT), EXCLUDING THE TIME FROM LEISURE ACTIVITY	
If you do this activity, is it an activity you wish no longer to do?	o Yes, I would like to stop doing this activity.<br>o No
If external assistance is provided, what type of professional provides the assistance?	o House keeper<br>o Other: ……………………………………….
ORGANISATION AND FOLLOW-UP OF THE PROFESSIONAL ASSISTANCE RECEIVED<br>Making appointments, communicating with professionals inside the home (e.g. nursing care, home help, ...) or outside the home (e.g. activities in day care centers or coordination of meetings/appointments with professionals)	
If you do this activity, is it an activity you wish no longer to do?	o Yes, I would like to stop doing this activity.<br>o No
If external assistance is provided, what type of professional provides the assistance?	o Care coordinator<br>o House keeper<br>o Other: ……………………………………….
MEDICAL TREATMENTS<br>TAKING MEDICINES : PILLS, INJECTIONS, ...<br>OTHER CARE: WOUND CARE, USE OF MEDICAL EQUIPMENT, PHYSIOTHERAPY OR MASSAGE	
If you do this activity, is it an activity you wish no longer to do?	o Yes, I would like to stop doing this activity.<br>o No
If external assistance is provided, what type of professional provides the assistance?	o Nurse<br>o Other: ……………………………………….

18. Here is a table showing the possible help you can provide to your family member, please fill it in.

**Activity carried out**	**Activity you wish no longer to do**	**If external assistance is provided, what type of professional aids?** (leave the box blank if no professional help is provided)
**Participation in training courses for carers**<br>*Any training related to the role of carer, whether in relation to support activities or in relation to a particular disease (e.g. Alzheimer's)*		
o Yes o No	o Yes o No	
**Finding the person you are helping who has run away**		
o Yes o No	o Yes o No	
**Supervision of the person being helped**<br>*If there is a risk to the person themselves or to others: communication with the person being helped to remind/explain the things to be done (including night-time supervision)*		
o Yes o No	o Yes o No	○ House keeper<br>○ Patient's warden<br>○ Other : ……………………………
**Care supervision and professional services**<br>*This activity does not mean a need to talk with professionals (already mentioned in "organisation and follow-up of professional helpers") but the need to check that everything is going well with these helpers, to ensure the quality/coherence of care, respect for the person being helped...*		
o Yes o No	o Yes o No	

19. In general, does your relative agree to be helped?

- ○ Yes
- ○ No

20. Does your relative have difficulty accepting help from professionals who come (e.g. he or she does not open the door, etc.)?

- ○ No
- ○ Yes, but not on a regular basis
- ○ Yes, frequently

21. **Does your relative have memory problems?**

- o Yes
- o No

22. **Does your family member have behavioral problems, for example, inappropriate social behavior (e.g. shouting, noise, rummaging through other people's things, etc.), wandering, verbal or physical aggression or inappropriate sexual behavior?**

- o Yes
- o No

23. Does your loved one have mood disorders, such as irritability, depression or paranoia, that make it difficult for you to help him or her?

- o Yes
- o No

24. Does caring for your loved one bring you satisfaction (satisfaction of helping others)?

- o Yes
- o No

25. If you had a choice, would you prefer your loved one to be in a nursing home?

- o Yes
- o No

26. Do you find it difficult to take time off work because of the help you provide for your loved one?

- o Yes
- o No

27. For each subsequent question, please circle one answer only.

**Do you manage to take time for yourself during the week (e.g. participate in leisure activities, see friends, rest)?**				
Never	From time to time but not enough	Often enough		
**Do you sometimes have to interrupt your activities unexpectedly during the day to take care of your loved one?**				
Never	Several times a week	Every day	Several times a day	
**Do you sometimes have to be interrupted unexpectedly at night to take care of your loved one?**				
Never	Several times a week	Every night	Several times a night	
**Do you feel that you do not have enough time for yourself because of the time you spend with your loved one?**				
Never	Rarely	Sometimes	Often enough	Almost always
**Do you feel stressed because you have to take care of your family member and at the same time try to cope with other family or work responsibilities?**				
Never	Rarely	Sometimes	Often enough	Almost always
**Do you feel anger towards your loved one when you are with him/her?**				
Never	Rarely	Sometimes	Often enough	Almost always
**Do you feel that your loved one is currently disrupting your relationships with other family members or friends?**				
Never	Rarely	Sometimes	Often enough	Almost always
**Do you feel stressed when you are with your relative?**				
Never	Rarely	Sometimes	Often enough	Almost always
**Do you feel that your health has deteriorated because you are caring for your relative?**				

Never	Rarely	Sometimes	Often enough	Almost always
**Do you feel that you do not have as much time for yourself and your other loved ones as you would like because of your loved one?**				
Never	Rarely	Sometimes	Often enough	Almost always
**Do you feel that your social life has deteriorated because you are taking care of your loved one?**				
Never	Rarely	Sometimes	Often enough	Almost always

28. How do you rate your health compared to other people of the same age?

- Better
- As good
- Poorer

29. **Have you had to stop or reduce your working hours to care for your family member?**

- Yes
- No

30. Do you need to help your loved one financially?

- Yes
- No

31. **Do you contribute to pay the costs related to your relative's loss of independence (for example for a household help, transport costs, medical expenses, etc.)?**

- Yes
- No

If yes, how much do you estimate this financial contribution per month (in Euros)?

32. Is your relative a woman or a man?

- A woman
- A man

33. **How old is your relative?** Answer:………………

34. If you would like to add comments to this questionnaire, please note them below:

Thank you very much for your participation!

The Geriatric Day Hospital Research Team
